# The increase in SARS-CoV-2 lineages during 2020–2022 in a state in the Brazilian Northeast is associated with a number of cases

**DOI:** 10.3389/fpubh.2023.1222152

**Published:** 2023-12-14

**Authors:** Moises Thiago de Souza Freitas, Ludmila Oliveira Carvalho Sena, Kiyoshi Ferreira Fukutani, Cliomar Alves dos Santos, Francisco das Chagas Barros Neto, Julienne Sousa Ribeiro, Erica Santos dos Reis, Valdir de Queiroz Balbino, Sérgio de Sá Paiva Leitão, Marcus Vinicius de Aragão Batista, Michael Wheeler Lipscomb, Tatiana Rodrigues de Moura

**Affiliations:** ^1^Health Sciences Graduate Program, Federal University of Sergipe, Aracaju, Brazil; ^2^Parasitic Biology Graduate Program, Federal University of Sergipe, São Cristóvão, Brazil; ^3^Health Foundation Parreiras Horta, Central Laboratory of Public Health (LACEN/SE), Sergipe State Health Secretariat, Aracaju, Brazil; ^4^Department of Microbiology and Immunology, Geisel School of Medicine at Dartmouth, Hanover, NH, United States; ^5^Center for Biological and Health Sciences, Federal University of Sergipe, São Cristóvão, Brazil; ^6^Department of Genetics, Federal University of Pernambuco, Recife, Brazil; ^7^Academic Unit of Serra Talhada, Rural Federal University of Pernambuco, Serra Talhada, Brazil; ^8^Department of Pharmacology, University of Minnesota, Minneapolis, MN, United States

**Keywords:** phylogenetic analysis, SARS-CoV-2 genomes, epidemiology, genomic surveillance, Sergipe

## Abstract

SARS-CoV-2 has caused a high number of deaths in several countries. In Brazil, there were 37 million confirmed cases of COVID-19 and 700,000 deaths caused by the disease. The population size and heterogeneity of the Brazilian population should be considered in epidemiological surveillance due to the varied tropism of the virus. As such, municipalities and states must be factored in for their unique specificities, such as socioeconomic conditions and population distribution. Here, we investigate the spatiotemporal dispersion of emerging SARS-CoV-2 lineages and their dynamics in each microregion from Sergipe state, northeastern Brazil, in the first 3 years of the pandemic. We analyzed 586 genomes sequenced between March 2020 and November 2022 extracted from the GISAID database. Phylogenetic analyses were carried out for each data set to reconstruct evolutionary history. Finally, the existence of a correlation between the number of lineages and infection cases by SARS-CoV-2 was evaluated. Aracaju, the largest city in northeastern Brazil, had the highest number of samples sequenced. This represented 54.6% (320) of the genomes, and consequently, the largest number of lineages identified. Studies also analyzed the relationship between mean lineage distributions and mean monthly infections, daily cases, daily deaths, and hospitalizations of vaccinated and unvaccinated patients. For this, a correlation matrix was created. Results revealed that the increase in the average number of SARS-CoV-2 variants was related to the average number of SARS-CoV-2 cases in both unvaccinated and vaccinated individuals. Thus, our data indicate that it is necessary to maintain epidemiological surveillance, especially in capital cities, since they have a high rate of circulation of resident and non-resident inhabitants, which contributes to the dynamics of the virus.

## Introduction

Severe acute respiratory syndrome coronavirus 2 (SARS-CoV-2) emerged in China in late 2019 and rapidly spread across the globe, leading the World Health Organization (WHO) to declare a pandemic state on 11 March 2020 (1, 2). The virus has been widespread, causing waves of infections in almost all regions of the world (3). The first cases were confirmed in the state of São Paulo in February 2020. After that, actions were taken by the Ministry of Health in order to contain the emerging epidemic (4). As of today, 37.9 million cases in Brazil have resulted in 706,531 deaths, representing a mortality rate of 441.3 individuals per 100,000 inhabitants (accessed on 28 October 2023; available in https://covid.saude.gov.br//). This high mortality rate is related to the lack of a national policy against the disease, the increasing population mobility, especially in large urban centers, the return of face-to-face work activities, difficulties in implementing individual and community preventive measures to reduce the spread of COVID-19, and delays in vaccination have contributed to the emergence and spread of SARS-CoV-2 variants of concern (VOCs) across the country over time (5).

In Brazil, the pandemic was characterized by the co-circulation of multiple variants over time (6). The emergence of new variants was directly related to adaptive mutations in the viral genome that modified the pathogenic potential of SARS-CoV-2. A single amino acid change can dramatically affect a virus’s ability to evade the immune system and complicate the clinical status of infected individuals (7). Alpha (B.1.1.7), Beta (B.1.351), Gamma (P.1), Delta (B.1.617.2), and omicron (B.1.1.529) lineages were important variants associated with greater transmissibility or virulence, reduced neutralization by antibodies obtained through natural infection or vaccination, ability to avoid detection, and/or decreased therapeutic or vaccination efficacy (8).

Monitoring SARS-CoV-2 was possible due to recent technological and scientific advances in genome sequencing and bioinformatics tools, allowing almost real-time genomic surveillance and tracking the emergence and replacement dynamics of variant emergence and prevalence among populations (9). Several studies focusing on genomic surveillance have provided crucial information to understand the dynamics of SARS-CoV-2 lineages in the states or regions of Brazil due to the large differences between inter- and intra-state population sizes, concentration, and dynamics of human movement (10, 11). This proposal has been shown to be relevant to determine the spread of the virus based on the specific characteristics of the state in a refined resolution (11). In the Brazilian Northeast, 7.4 million cases of COVID-19 and 136,000 deaths have already been reported. Bahia, Ceará, and Pernambuco are the states with the highest incidence of cases and deaths in the region. In Sergipe state, 363,329 individuals were diagnosed with COVID-19, resulting in 6,539 deaths (accessed on 28 October 2023; available at https://covid.saude.gov.br//). At the moment, a single study has been identified in the literature related to genomic surveillance in Sergipe, and this analyzed genomes sequenced between March 2020 and February 2021 (5). This demonstrates the necessity to implement new research aimed at understanding the effects of the pandemic.

Therefore, this study aimed to assess the dynamics of SARS-CoV-2 variants from 2020 to 2022 in the state of Sergipe within Brazil. Knowledge gained would identify viral evolutionary patterns and behavior as it relates to epidemiological impacts.

## Methods

### Study area

**Figure 1:**
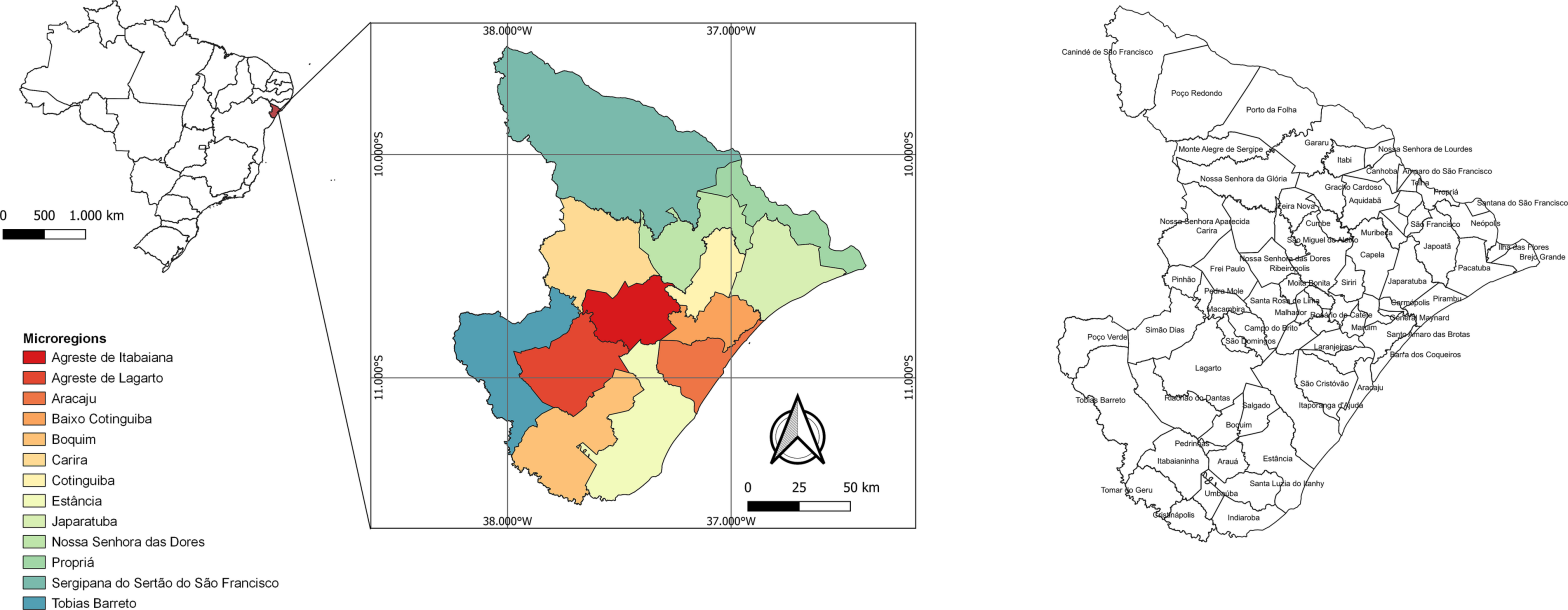
State map of Brazil with emphasis on Sergipe state, showing the 13 microregions.

Sergipe is located in northeastern Brazil and has a land area of 21,938,188 km^2^ and an estimated population of 2,338,474 inhabitants. The state has 75 municipalities and is divided into 13 microregions (Agreste de Itabaiana, Agreste de Lagarto, Aracaju, Baixo Cotinguiba, Boquim, Carira, Cotinguiba, Estância, Japaratuba, Nossa Senhora das Dores, Propriá, Sergipana do Sertão do São Francisco, and Tobias Barreto) (Figure 1). The microregion of Aracaju is made up of the capital (Aracaju), and the municipalities of Barra dos Coqueiros, Nossa Senhora do Socorro, and São Cristóvão, forming the metropolitan region of Aracaju, which represents approximately 36% of the state population^1^.

### Data collection

Full-length SARS-CoV-2 genomes from February 2020 to November 2022 were obtained from the GISAID database^2^. Only complete genomes and complete collection data were used. The sequences were evaluated individually, considering the lineage, which was determined by the Pangolin software^3^, municipality, and collection date. Soon after, the genomes were separated by year, giving rise to three data sets. The Circos program (12) was used to visualize the distribution of the genomes by strains and municipalities.

In order to correlate the number of lineages of SARS-CoV-2 with the average of infections by months, daily cases, daily deaths, and admissions of vaccinated and unvaccinated patients, the data were uploaded to a cross-country database of COVID-19^4^ (13, 14). The Pearson correlation test was performed using a native stats (V.4.0.3) package available in R software, and the grouped stacked bars with the abundance of lineage between months were performed and represented using the ggplot2 package (15, 16) and the correlation matrix was performed using *corrplot* package (17). All the differences with *p*-values <0.05 were considered statistically significant.

### Phylogenetic analyses

Multiple sequence alignment was carried out using MAFFT v.7 with auto and add fragments parameters (18). The sequence from Wuhan-Hu-1 (NC_045512.2) was then added as an outgroup. Subsequently, the maximum likelihood (ML) phylogenetic trees were built using IQ-TREE v2.1.2 (19). The nucleotide substitution models TN + F, GTR + F + I + I + R4, and TIM + F + I + I + R3 were selected using ModelFinder in IQ-TREE2 v2.1.2 for the SARS-CoV-2 genomes of 2020, 2021, and 2022, respectively (20). Clade support was estimated using 1,000 replicates of bootstrap. The tree was visualized and edited using the iTOL v.4 tool (21). The haplotype network was created with PopART software version 1.7 (22) using the median-joining method to identify the existence of shared haplotypes.

### Spatial analysis

The maps to represent the spatial distribution of SARS-CoV-2 lineages were constructed using the QGIS software version 3.18.2, with the cartographic projection corresponding to the Universal Reference System SIRGAS 2000. The cartographic projection used corresponded to the Universal Transverse Mercator (UTM) system, Terra Datum horizontal model (SIRGAS 2000) to segment by municipalities and states were collected from the databases of the Brazilian Institute of Geography and Statistics (IBGE).

## Results

### Genomic surveillance of SARS-CoV-2 variants in Sergipe

**Figure 2:**
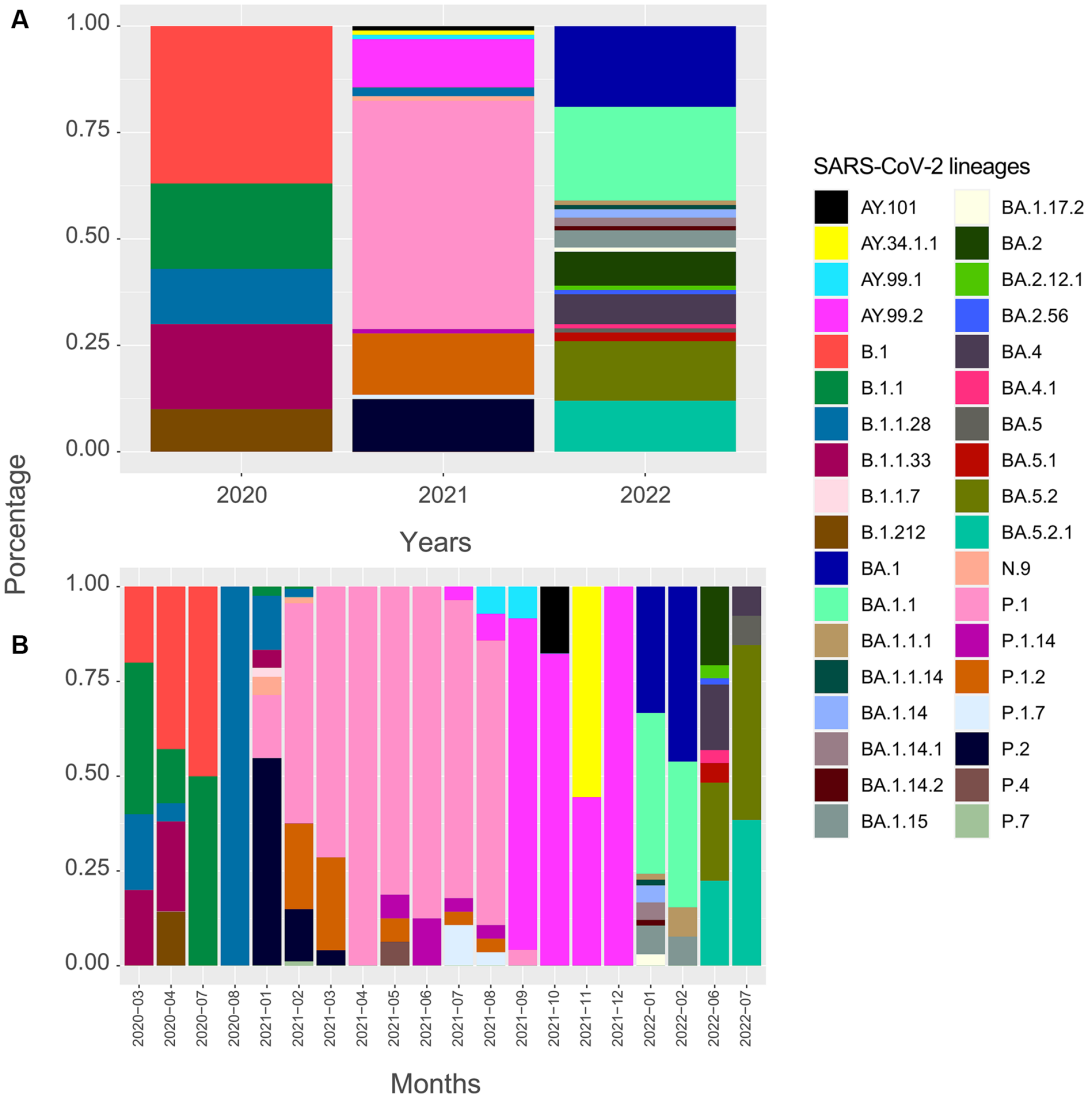
Timeline of genomes sequenced for the SARS-CoV-2 virus in Sergipe state, Brazil (SARS-CoV-2 lineages in the state from March 2020 to November 2022, obtained from GISAID database).

For this analysis, 586 SARS-CoV-2 viral genomes were evaluated and classified into 36 variant lineages (Figure 2). Sequences have been distributed in 47 municipalities, representing 62.7% of the total. Most of the genomes obtained from the GISAID database have their origin in the Aracaju microregion, as can be seen in Table 1.

**Table 1:** Distribution of SARS-CoV-2 genomes obtained from the GISAID database divided by microregions of Sergipe.

Microregions	Municipalities	2020	2021	2022	%
Aracaju	Aracaju	19	252	49	69.6%
	Barra dos Coqueiros	02	11	22	
	São Cristovão	–	02	12	
	Nossa Senhora do Socorro	01	25	13	
Estância	Estância	01	05	–	2.6%
	Itaporanga D’ajuda	01	07	01	
Agreste de Lagarto	Lagarto	01	01	05	2.2%
	Riachão do Dantas	–	02	04	
Baixo Cotinguiba	Laranjeiras	01	04	–	1.9%
	Riachuelo	01	01	–	
	Maruim	–	01	–	
	Santo Amaro das Brotas	–	01	–	
	Carmópolis	–	–	02	
Propriá	Telha	01	01	–	1.9%
	Amparo de São Francisco	–	01	–	
	Canhoba	–	01	–	
	Cedro de São João	–	03	–	
	Nossa Senhora de Lourdes	–	01	01	
	Propriá	–	02	–	
Boquim	Tomar do Geru	01	02	–	4.4%
	Boquim	–	06	02	
	Cristinápolis	–	02	–	
	Itabaianinha	–	06	–	
	Salgado	–	03	03	
	Umbaúba	–	–	01	
Nossa Senhora das Dores	Aquidabã	–	02	–	0.5%
	Nossa Senhora das Dores	–	–	01	
Sergipana do Sertão do São Francisco	Canindé de São Francisco	–	14	–	4.8%
	Nossa Senhora da Glória	01	05	–	
	Gararu	–	01	–	
	Monte Alegre de Sergipe	–	03	–	
	Porto da Folha	–	04	–	
Cotinguiba	Capela	–	04	03	1.9%
	Divina Pastora	–	–	04	
Carira	Carira	–	01	–	3.4%
	Frei Paulo	–	03	03	
	Ribeirópolis	–	08	04	
	Pinhão	–	–	01	
Agreste de Itabaiana	Itabaiana	–	04	09	2.9%
	Areia Branca	–	–	02	
	Macambira	–	–	01	
	Malhador	–	–	01	
Tobias Barreto	Simão Dias	–	11	01	3.4%
	Tobias Barreto	–	04	02	
	Poço Verde	–	01	01	
Japaratuba	Pirambu	–	01	–	0.5%
	Japaratuba	–	–	02	
Total		30	406	150	

**Figure 3:**
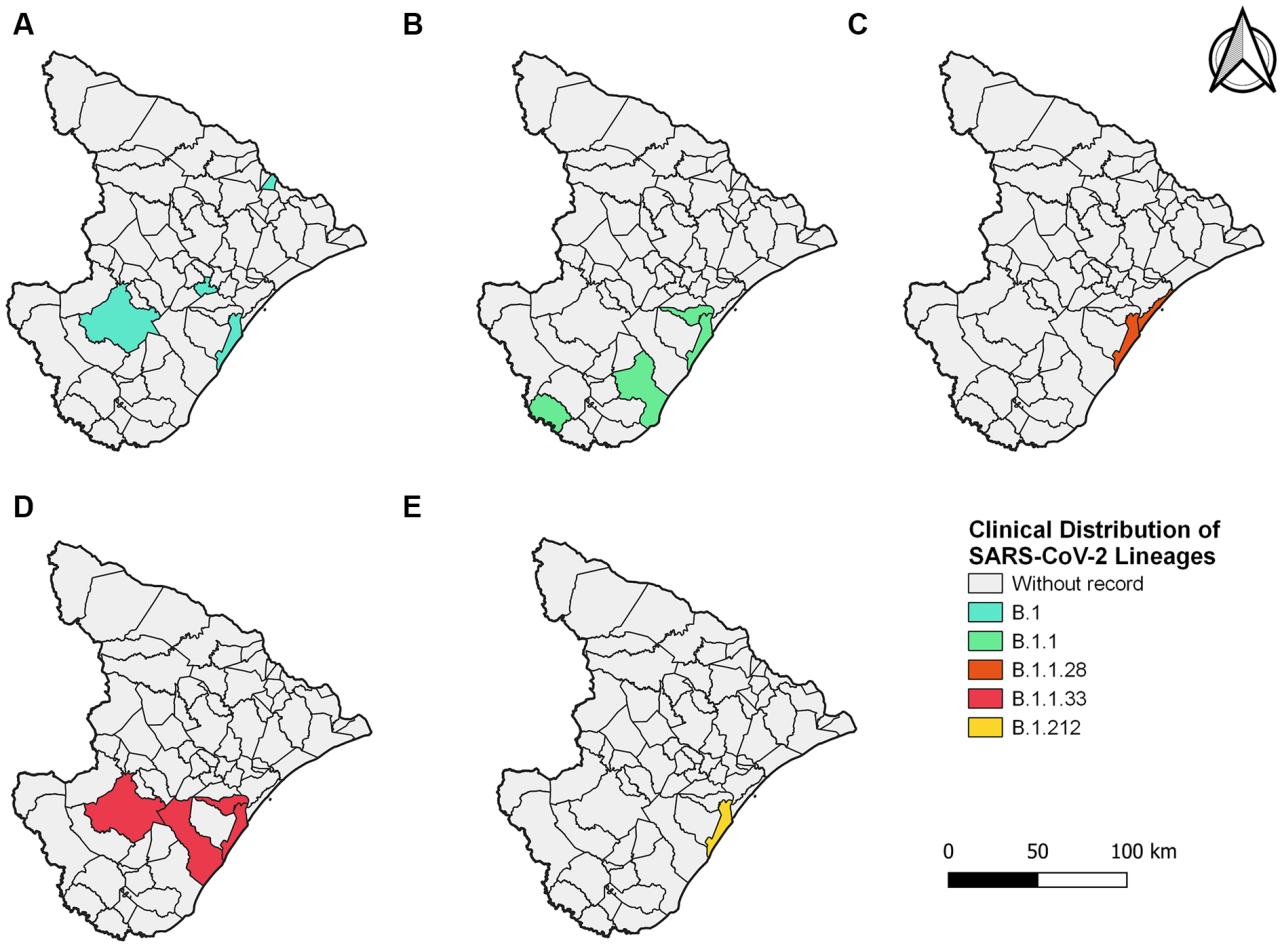
Sergipe state map showing the clinical distribution of the SARS-CoV-2 lineages in 2020 by municipality. Colors represent the municipality and lineages downloaded from the GISAID database.

In 2020, five lineages were detected circulating in Sergipe, B.1 (11 sequences, 36.7%) was the most frequent, followed by B.1.1 (6 sequences, 20%), B.1.1.33 (6 sequences, 20%), B.1.1.28 (4 sequences, 13.3%), and B.1.212 (3 sequences, 10%) (Figure 2). A total of 30 genomes were available on the GISAID database. In total, 19 of those 30 genomes were related to samples from Aracaju (Supplementary Figure S1). Genomic sequences have also been observed in 10 other municipalities (Figure 3). At first, B.1 was identified in the state on 12 March 2020 during the first wave. This sample belongs to an individual who resided in Aracaju with a travel history to Europe (Spain).

**Figure 4:**
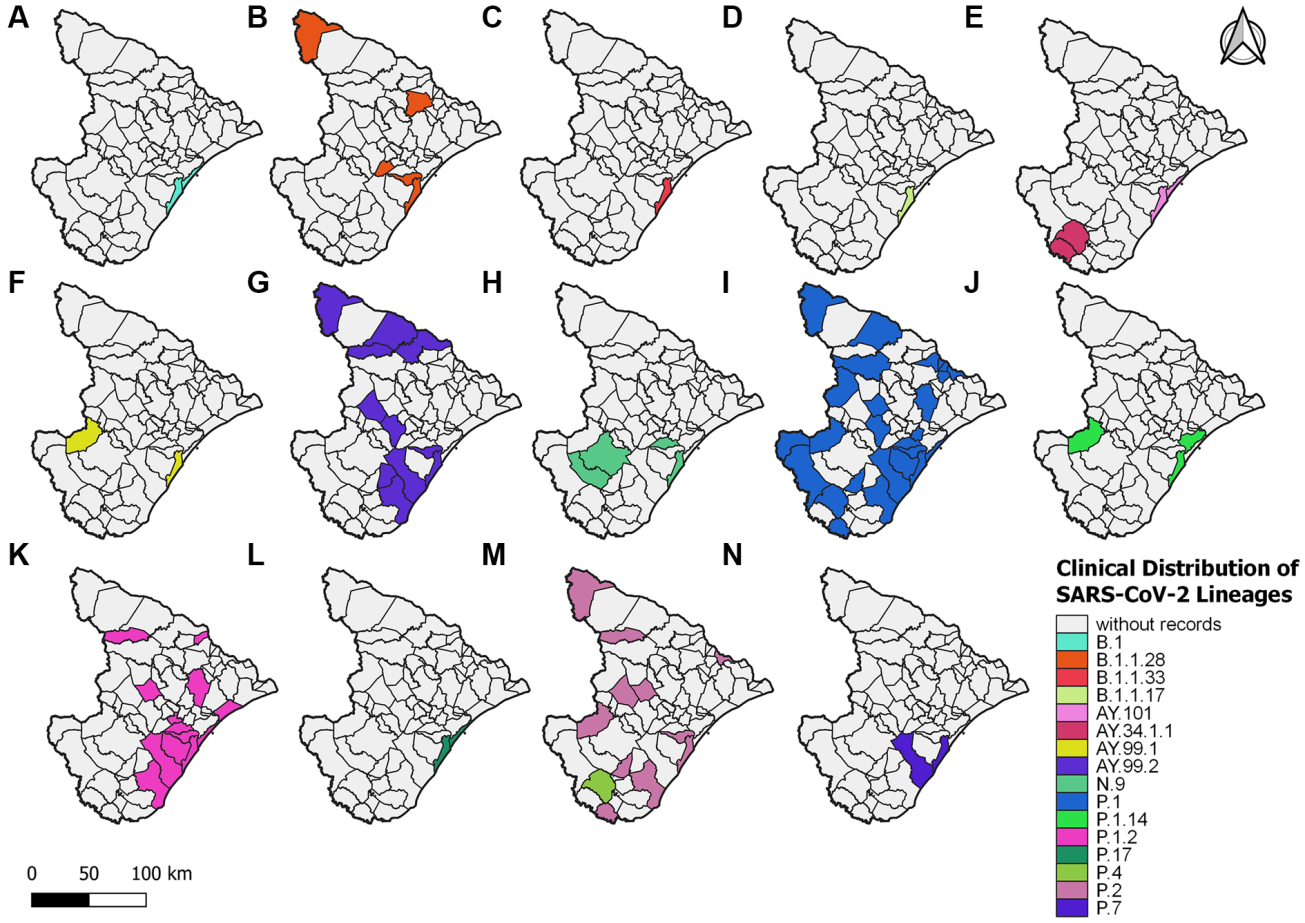
Sergipe state map showing the clinical distribution of the SARS-CoV-2 lineages in 2021 by municipality. Colors represent the municipality and lineages downloaded from the GISAID database.

For 2021, 406 sequences were used to create the datasets and subsequently classified into 16 viral variant lineages. In total, 212 samples were identified as the P.1 gamma variant, representing approximately 52.2% (Figure 2). Initially, the circulation of this variant was registered on 17 January 2021 in the municipality of Aracaju during the second wave. Delta sequences have been registered in Sergipe between January and September. P.1.2 (56 sequences, 13.8%) and P.2 Zeta variant (50 sequences, 12.3%) were also highly represented (Figure 4). This variant was predominant in infection cases from September and December. A total of 57 genomes of the AY.* lineages were found in the GISAID database. This is distributed in four strains (AY.34.1.1, AY.99.1, AY.99.2, and AY.101). AY.99.2 (45 sequences, 11.1%) was prevalent during this period. Lineages AY.34.1.1, AY.99.1, and AY.101 represented approximately 2.9% of the total genomes (Figure 2). All other strains identified in 2021 represent approximately 10.6% (43 sequences). Aracaju was the municipality with the highest number of strains circulating when compared to other localities (Figure 4). Lineages were also identified in 37 other cities (Supplementary Figure S2).

**Figure 5:**
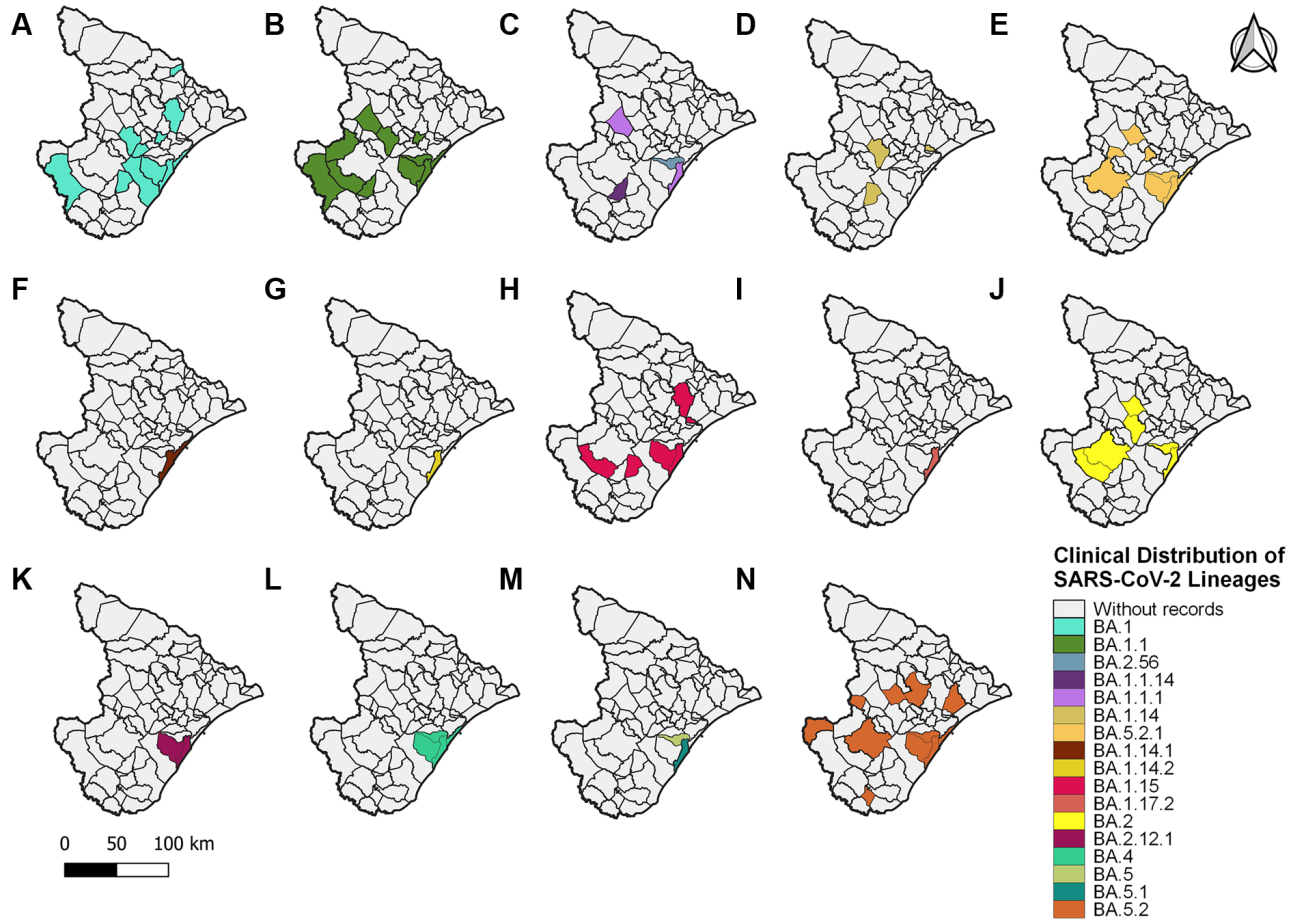
Sergipe state map showing the clinical distribution of the SARS-CoV-2 lineages in 2022 by municipality. Colors represent the municipality and lineages downloaded from the GISAID database.

An alignment with 150 genomes was created using the genomes of 2022, and it was possible to identify 18 lineages distributed in 27 municipalities (Figure 5) (Supplementary Figure S3). In January, nine sublineages of the Omicron variant were identified as circulating. The first variant sample detected was on 3 January 2022. The lineage BA.1.1 (33 sequences, 22%) was the most frequent during the third wave, followed by BA.1 (28 sequences, 18.7%), BA.5.2 (21 sequences, 14%), and BA.5.2.1 (18 sequences, 12%). All other lineages identified represented approximately 33.3% (50 sequences) (Figure 2).

### Evolutionary analysis of SARS-CoV-2 lineages

**Figure 6:**
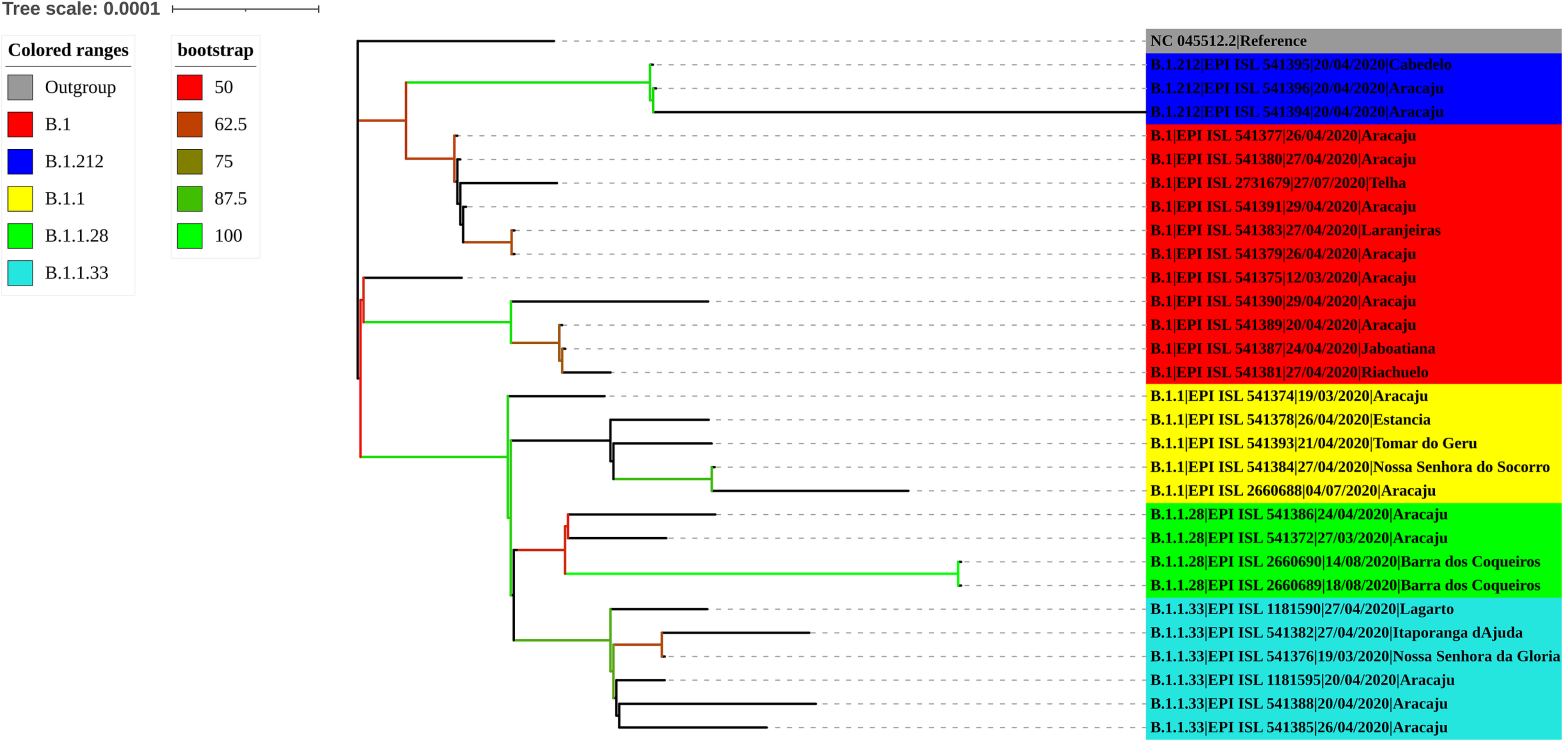
Maximum likelihood tree of 30 SARS-CoV-2 whole-genome sequences available at the GISAID database using the TN + F evolutive model. The colors assigned to the branches are related to their respective lineages and the samples are labeled according to their sampling dates and virus lineages.

**Figure 7:**
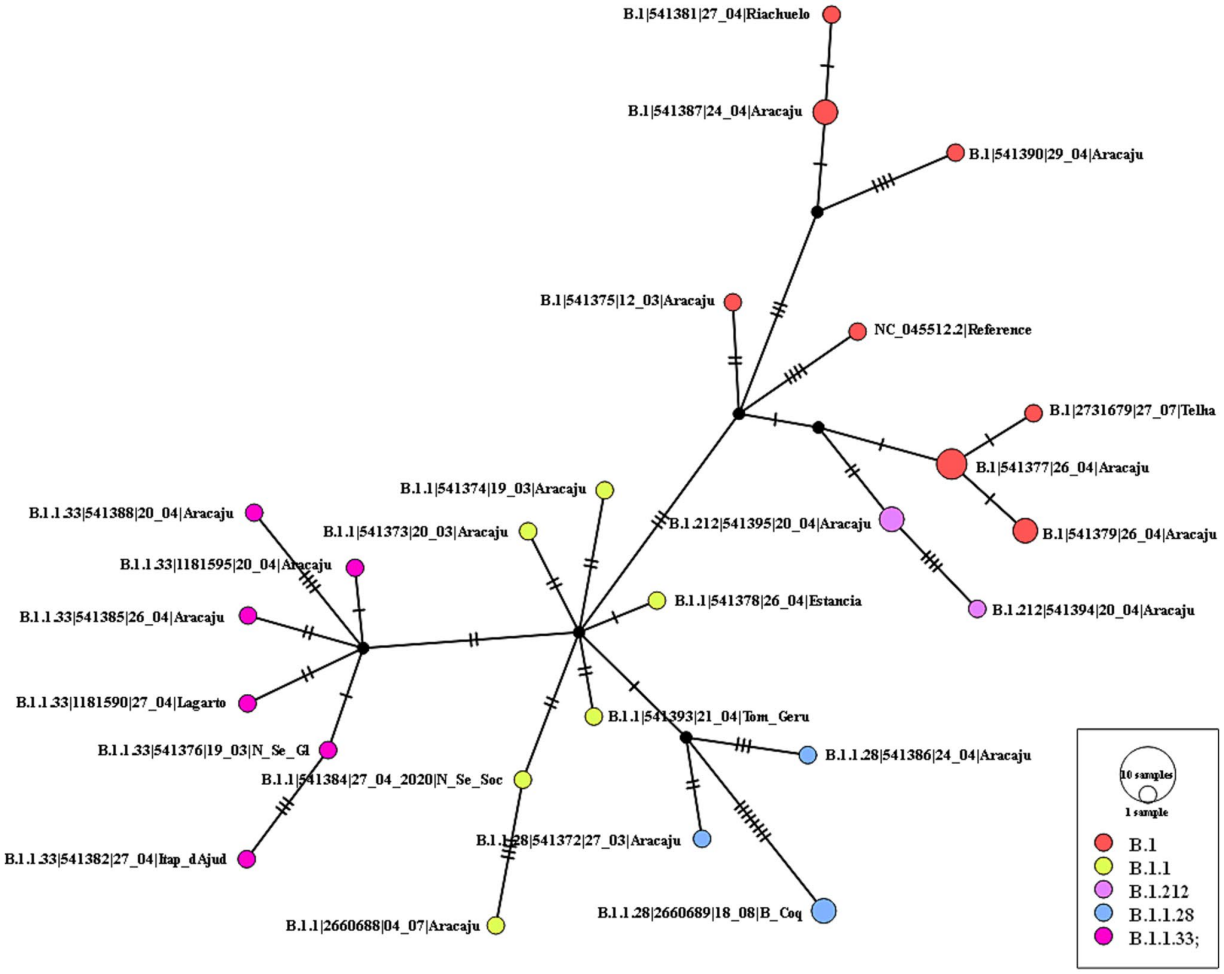
Haplotype network, obtained with PopART software, showing relationships among haplotypes of the SARS-CoV-2 genomes available in 2020. Each circle represents one haplotype. Diameters of the circles correspond to the frequencies of the respective haplotypes. The numbers of dashes display mutational steps (each dash stands for one single nucleotide mutation). Small black circles represent hypothetical (missing) haplotypes.

The maximum likelihood phylogenetic tree was constructed to confirm the SARS-CoV-2 variant classification that circulated between February 2020 and November 2022 in the state of Sergipe. Considering the sequences from 2020, the phylogenetic analysis suggested five distinct well-supported groups (B.1, B.1.1, B.1.212, B.1.1.28, and B.1.1.33) (Figure 6). The haplotype network has been constructed with the purpose of characterizing the ancestral relationships maintained between the lineages. Five well-defined clades (B.1, B.1.1, B.1.212, B.1.1.28, and B.1.1.33) were identified. Notably, haplotype sharing was not observed among the sequences from these different strains (Figure 7).

**Figure 8:**
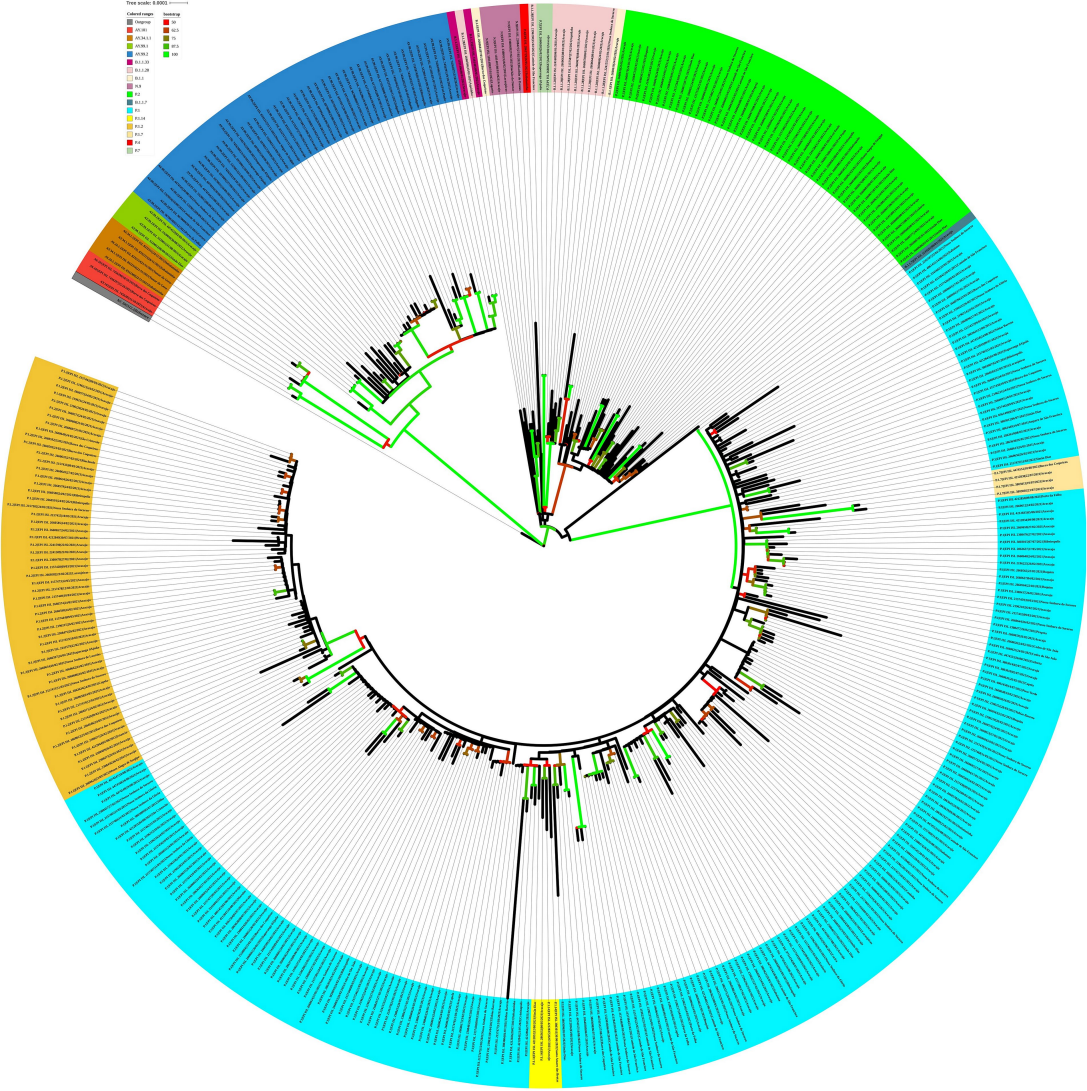
Maximum likelihood tree of 406 SARS-CoV-2 whole-genome sequences available at the GISAID database using the GTR + F + I + I + R4 evolutive model. The colors assigned to the branches are related to their respective lineages and the samples are labeled according to their sampling dates and virus lineages.

**Figure 9:**
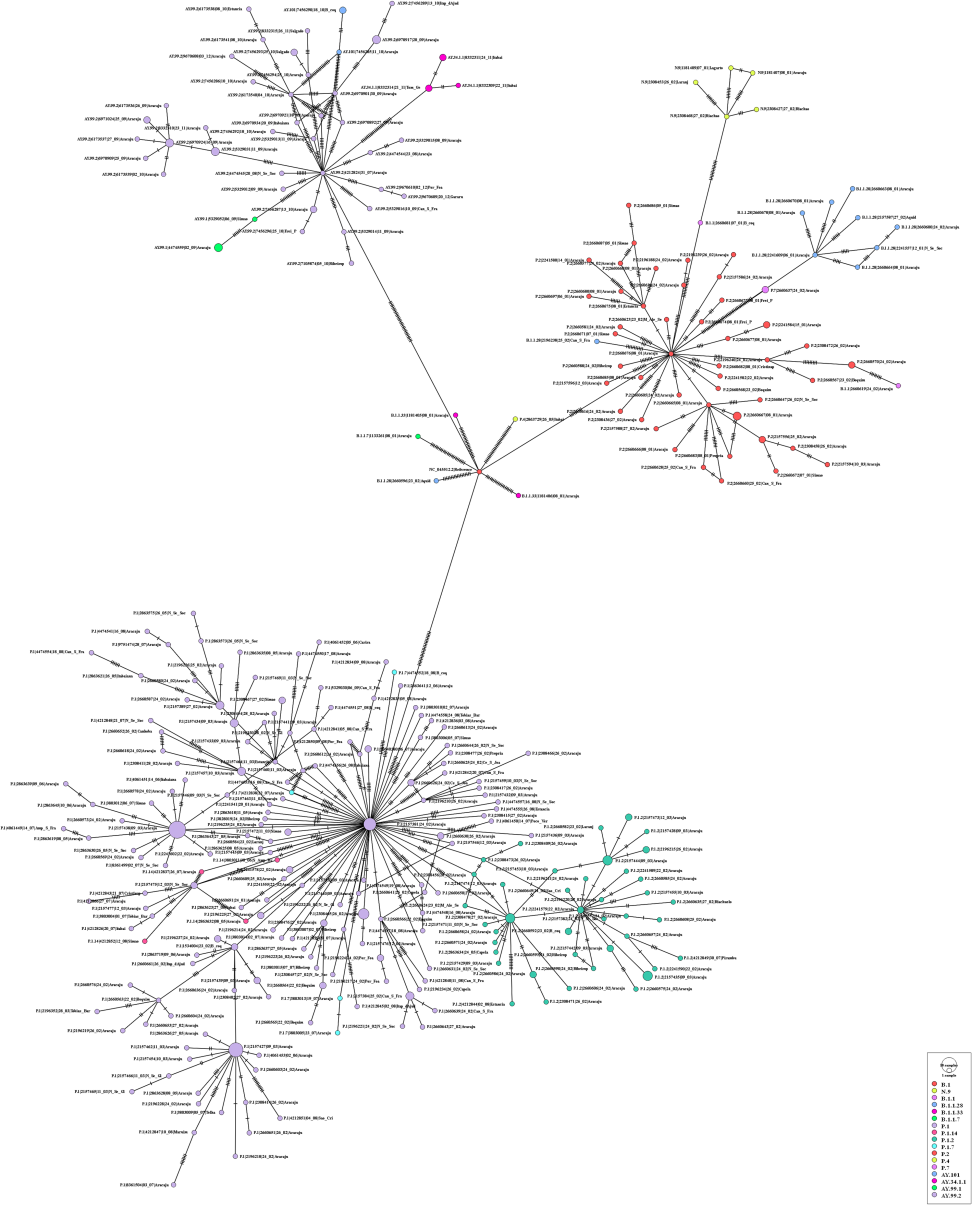
Haplotype network, obtained with PopART software, showing relationships among haplotypes of the SARS-CoV-2 genomes available in 2021. Each circle represents one haplotype. Diameters of the circles correspond to the frequencies of the respective haplotypes. The numbers of dashes display mutational steps (each dash stands for one single nucleotide mutation). Small black circles represent hypothetical (missing) haplotypes.

For the genomes from 2021, the ML tree revealed five main well-supported clades. One clade was composed only of the delta variant. N.9, B.1.1.28, and P.2 lineages were divided into different clades with significant support values (Figure 8). B.1.1.28 was identified as a common ancestor of P.1 and P.2. A clade represented by sublineages relative to P.1 (P.1.7, P.1.14, and P.1.2) was observed. However, sequences belonging to lineages B.1.1, B.1.1.33, and P.4 have not demonstrated significant bootstrap values. P.7 clade showed high support value, and its genetic pattern is associated with the P.2 lineage. The haplotype network revealed five heterogeneous clusters, where P.1 was associated with P.1.2, P.1.14, and P.1.7. Another cluster was observed with P.2 and P.7 lineages. N.9, B.1.1.28, and all AY.* remained isolated in the phylogenetic tree (Figure 9).

**Figure 10:**
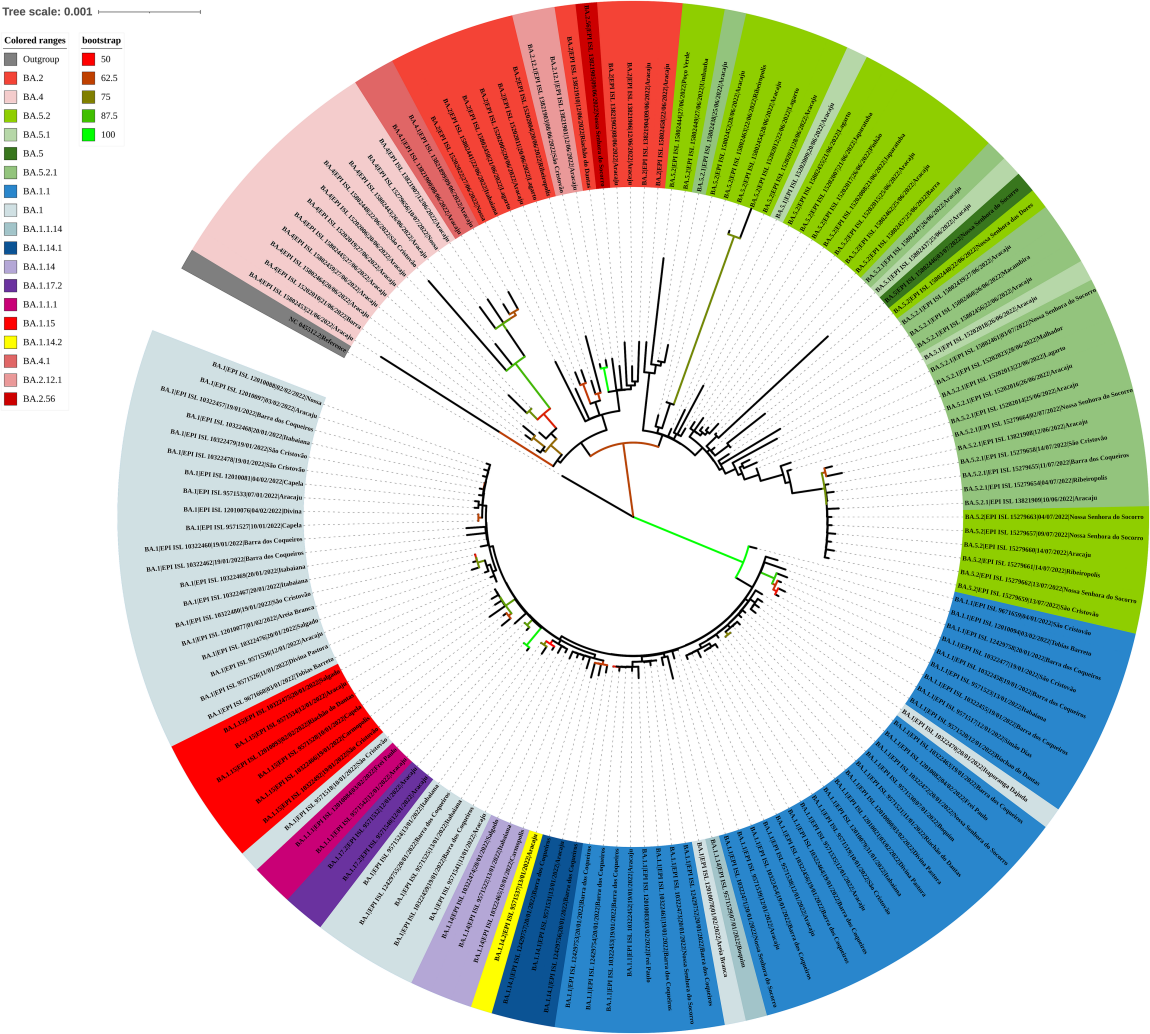
Maximum likelihood tree of 150 SARS-CoV-2 whole-genome sequences available at the GISAID database using the TIM + F + I + I + R3 evolutive model. The colors assigned to the branches are related to their respective lineages and the samples are labeled according to their sampling dates and virus lineages.

**Figure 11:**
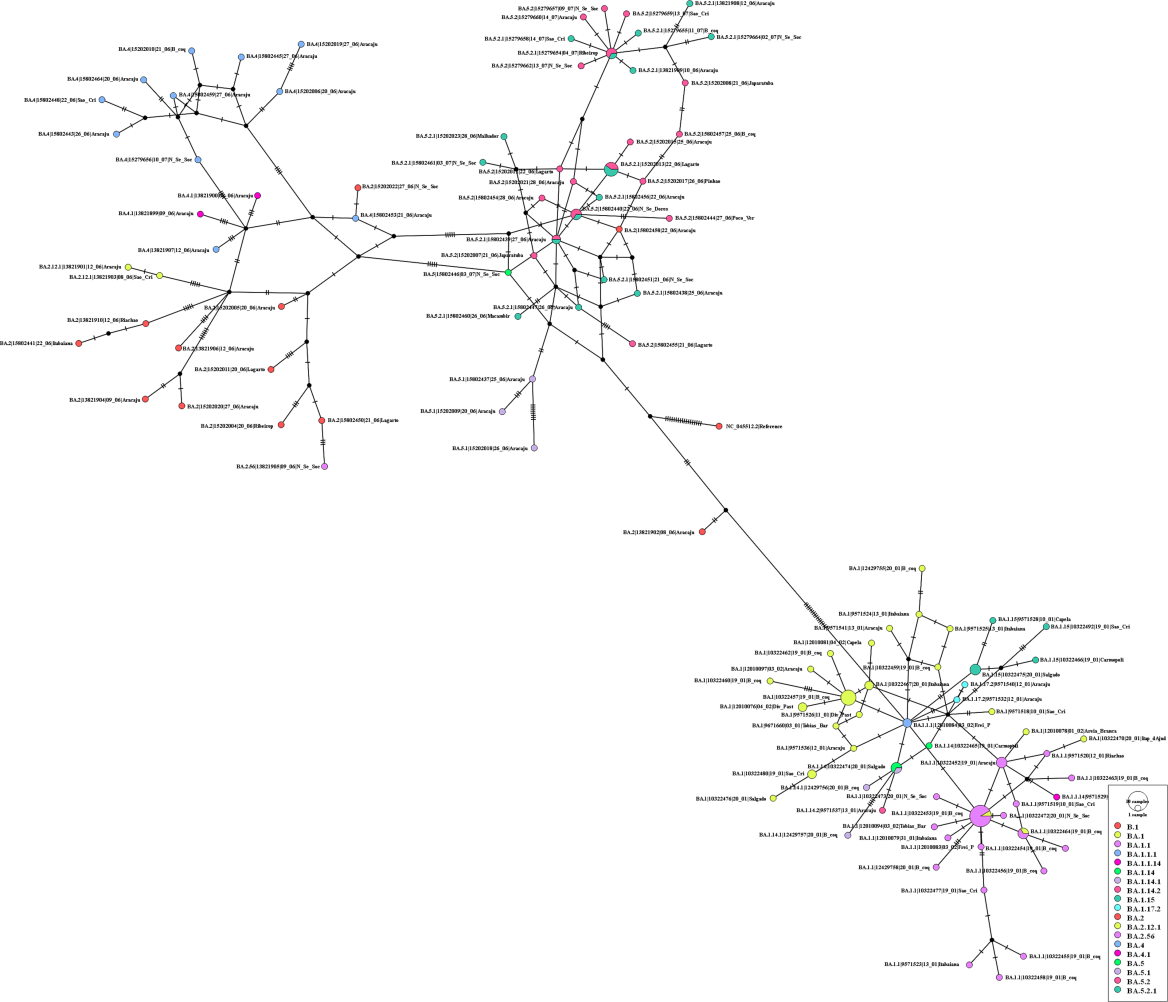
Haplotype network, obtained with PopART software, showing relationships among haplotypes of the SARS-CoV-2 genomes available in 2022. Each circle represents one haplotype. Diameters of the circles correspond to the frequencies of the respective haplotypes. The numbers of dashes display mutational steps (each dash stands for one single nucleotide mutation). Small black circles represent hypothetical (missing) haplotypes.

Analyzing the genomes from 2022, it was possible to observe differences, revealing two distinct clades (Figure 10); both groups were significantly supported. A clade was formed by genomes from lineages BA.5, BA.5.1, BA.5.2, BA.5.2.1, BA.2, BA.2.12.1, BA.2.56, BA.4, and BA.4.1, and another clade was formed by genomes from lineages BA.1, BA.1.1, BA.1.14, BA.1.14.1, BA.1.14.2, BA.1.15, BA.1.17.2, BA.1.1.1, and BA.1.1.14. The haplotype network also suggested two clusters. One cluster was composed by BA.5, BA.5.1, BA.5.2, BA.5.2.1, BA.2, BA.2.12.1, BA.2.56, BA.4, and BA.4.1. On the other hand, the other cluster was formed by lineages BA.1, BA.1.1, BA.1.14, BA.1.14.1, BA.1.14.2, BA.1.15, BA.1.17.2, BA.1.1.1, and BA.1.1.14. In addition, it was observed genomes from different lineages sharing haplotypes, such as BA.1.1|10,322,472, BA.1.1|10,322,464, BA.1.14|10,322,474, BA.5.2.1|15,279,654, BA.5.2.1|15,202,013, BA.5.2.1|15,802,440, and BA.5.2.1|15,802,439 (Figure 11). In the BA.1.1|10,322,472 genome, genetic patterns associated with BA.1 and BA.1.1 have been identified, suggesting the maintenance of ancestral relationship.

### Correlation analysis of SARS-CoV-2 lineages and infection cases in Sergipe

**Figure 12:**
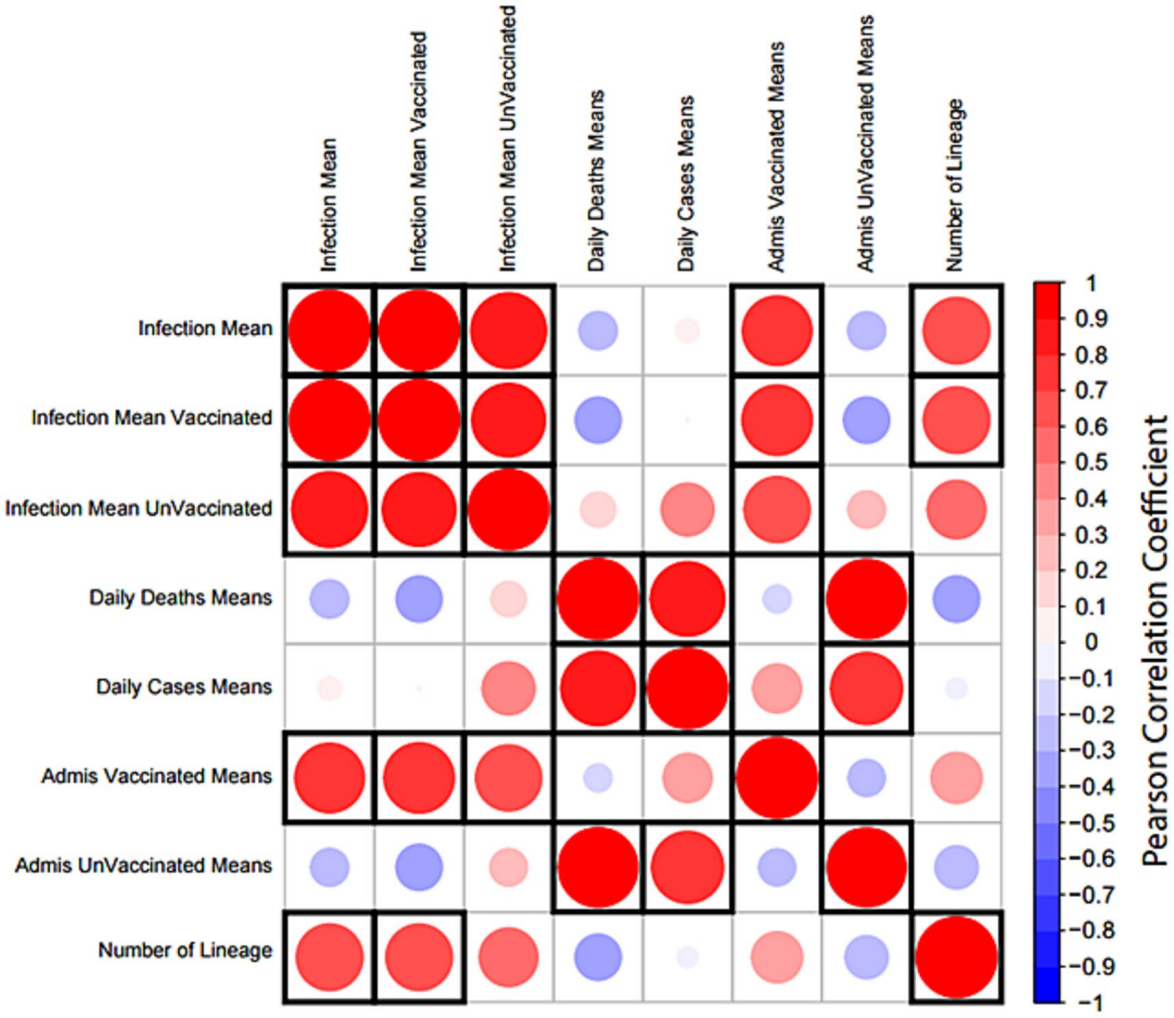
Correlation matrix analysis of epidemiological data and SARS-CoV-19 lineages. The distribution means of lineage and epidemiological data per month were obtained from a cross-country COVID-19 database. All variables were tested for correlation using the Pearson correlation test. The size and color of each circle indicate the strength of the correlation coefficient; red indicates a positive correlation and blue indicates a negative correlation. A significant correlation (*p*-value <0.05) is indicated by a black frame. The matrix was created using the corrplot package in R.

A correlation matrix analysis was employed to examine the relationship between the average distributions of viral lineages and the average number of individuals infected, as indicated by registered cases reported by global epidemics (Figure 12). Furthermore, we explored relationships among the averages of hospital admissions in vaccinated and unvaccinated patients as well as the averages of daily deaths and daily cases (Figure 12). Notably, the averages of registered cases exhibited a correlation cluster among all reported cases as well as between vaccinated and unvaccinated patients. Additionally, the averages of daily cases and deaths showed a direct correlation. Hospital admissions in vaccinated patients were associated with clusters of registered cases, while hospital admissions in unvaccinated patients were linked to daily deaths and daily cases. The observed increase in the number of variant lineages during the study period was directly correlated with the averages of infections.

## Discussion

This is a pioneering study of the state of Sergipe. The evolutionary history of circulating SARS-CoV-2 genomes over the last 3 years was reconstructed using phylogenetic analyses based on ML and the median-joining method. Our data support that B.1 was the first lineage detected in Sergipe (as recorded in Aracaju on 12 March 2020). However, Gurgel et al. (23) report in their study that the first case of COVID-19 in the state of Sergipe may have occurred a few months earlier, as samples from asymptomatic individuals sent for blood tests between the months of January and April 2020 by reasons unrelated to COVID-19 showed the presence of SARS-CoV-2 immunoglobulins (IgM and IgG) before the notification of clinical cases in the state. The country registered its first COVID-19 case in late February in São Paulo (4). In Bahia, the first confirmed SARS-CoV-2 infections occurred on 28 February, being the first case in the Northeast region^5^.

The lineages B.1, B.1.1, B.1.1.33, B.1.1.28, and B.1.212 were prevalent until mid-August 2020. In an analysis carried out by Dos Santos et al. (5), B.1 (58.5%), B.1.1.33 (17.1%), B.1.1.119 (12.2%), B.1.1.28 (9.8%), and B.1.212 (2.4%) were dominant from March to August 2020 in Sergipe. In late 2020, the variants zeta (P.2) (24) and gamma (P.1) (25), descendants of lineage variants B.1.1.28, emerged and were associated with the second phase of the pandemic. P.1 was identified in the state of Amazonas in mid-December 2020, with a proposed emergence around November (25, 26).

P.1 was dominant in infection cases by SARS-CoV-2, having their circulation on 17 January 2021. Some studies proposed that the emergence of three mutations E484K, N501Y, and K417T in the Spike protein allowed the virus to escape from the host immune response (27–29). In mid-January 2021, samples from 11 suspected cases and their contact reporting a travel history to/from Amazonas state were screened at the Central Laboratory of Health of the Bahia state (LACEN-BA). Genetic evidence has confirmed for the first time the circulation of the P.1 in the Brazilian Northeast (30). The study conducted by Dos Santos et al. (5) also confirms that P.1 circulated in Aracaju (Sergipe) on 17 January 2021 in a sample belonging to a resident from the city of Manaus (Amazonas) who traveled to Sergipe to visit his family (5).

Our results from the evolutionary analysis showed that B.1.1.28 is a common ancestor of P.1 and P.2. In the studies conducted by Varela et al. (31) and Harvey et al. (32), it was indicated that P.1 and P.2 descend from B.1.1.28 although they have different times of appearance and share the S: E484K mutation. A cluster formed by P.1, P.1.1, P.1.2, and P.1.7 demonstrates the ancestral relationship between these lineages, as reported by Varela et al. (31) and Machado et al. (11). In the present study, a shared ancestry between P.2 and P.7 was also observed similar to that suggested by Lamarca et al. (33).

Assessing the results, it was suggested that AY.99.2 (11.1%) became dominant in cases from September to December 2021. There are signs that the delta variant emerged in October 2020 on the Asian continent as has been classified by WHO (33). In Brazil, the first community-sustained transmission chains of the delta variant were registered in June 2021 in the state of Rio de Janeiro (34), and it has been widely detected in other Brazilian states over time (see footnote 5). Among the delta variants, the AY.99.2 was the most dominant, reaching 58% of all sublineages sampled during the period (35). Some evidence demonstrate that AY.99.2 emerged in Brazil; the first SARS-CoV-2 genomes from this lineage available in the GISAID database are from samples collected in April 2021 in the northeastern state of Ceará (36). Studies have identified mutations in the spike protein of the delta sublineages found in Brazil, and the most common mutations mentioned are T19R, T95I, E156G, DEL157/158, L452R, T478K, D614G, Q677H, P681R, D950N, V1104L, and L1265F^7^. Some of these mutations can be related to viral fitness advantages such as enhanced viral entry, pathogenesis, and immune escape (37, 38). Despite that, the number of hospitalizations declined from 6.9% (January to June 2021) to 3.6% (July to December 2021) during the community transmission of the delta variant with the progression of vaccination in the second half of 2021 in Sergipe (39).

The phylogenetic tree revealed that the samples of the delta variant formed a monophyletic clade. In an analysis using the neighbor-joining method with genomes from the delta lineage, a compatible structuring with a monophyletic clade was shown, and the omicron variant emerged from it (40).

In this study, the lineage BA.1.1 was reported as most frequent, followed by BA.1, BA.5.2, and BA.5.2.1. Genomic surveillance detected that in February 2022, the omicron variant was majority; 99.8% of the samples analyzed around the country being positive for the variant (see footnote 5). From January to September, BA.1 (4,253 genomes) and BA.1.1 (2,521 genomes) were also prevalent in infection cases by SARS-CoV-2 in the northeast Brazil (see footnote 5). Since the beginning of the pandemic, the SARS-CoV-2 genome has been rapidly evolving. This is mostly due to the inherent polymerase mistakes and host immune selection factors (32). The omicron variant is the most mutated variant containing more than 60 mutations in its genome. In total, 32 of these mutations lie within the receptor binding domain (RBD) of the spike protein (41, 42). The large number of mutations associated with the spike RBD domain can be related to infectivity rates, high transmission capacity, and rapid dispersal potential (26). Among the mutations identified in the omicron variants, 14 are exclusive and found in all the omicron variants (43).

Our phylogenetic tree for omicron VOC suggests the existence of two main clades, one composed of the sublineages linked to BA.1 and the other associated with BA.2, BA.4, and BA.5 variants. In a study developed by Veneziano et al. (44) with Omicron SARS-CoV-2 genomes in Italy, a group composed of BA.1 and another by BA.2, BA.4, and BA.5 lineages was identified. Six mutations have been identified in all the omicron variants, excluding omicron BA.1: Del24-26, V213G, T376A, S371F, D405N, and R408S (43). These mutations may have contributed to the structure of the clades of the phylogenetic tree and also in the haplotype network, as observed in other organisms (45, 46). Notably, our study presents a limitation due to the fact that in some months of 2020 and 2022, there were no records of SARS-CoV-2 genomes in the GISAID database. However, there is agreement between our genomic surveillance results and those observed in other states of Brazil.

Finally, the increase in the average number of SARS-CoV-2 lineages during the studied periods is related to the average number of infections in both unvaccinated and vaccinated individuals. Tarkowski et al. (47) demonstrated that vaccinated individuals presented higher levels of IgG against viral proteins of spike protein-1 (S1) and receptor-binding domain (RBD), which resulted in a better immune response to B.1 and P.1 variants although immune activation is less noticeable in response to the B.1.617.2 variant. A similar study revealed differences in the efficiency of humoral activity in vaccinated individuals against B.1.617.1, B.1.617.2, B.1.351, and P.1 lineages due to mutations in the spike protein (S) (48). Unvaccinated individuals are intrinsically associated with daily cases and deaths. Martins-Filho et al. (39) studied the dynamics of hospitalizations and the predominance of delta and omicron variants in the Northeast of Brazil and found that during the circulation of the delta variant (July to December 2021), the majority of deaths occurred in people who were not vaccinated or who had not completed the vaccination schedule. Furthermore, in 2020, vaccines were scarce, with high hospitalization rates (46% of the population with active infection) (39).

## Conclusion

Our data suggest a correlation between the increase in the mean number of variant lineage strains and the mean number of infections in unvaccinated and vaccinated individuals. It is important to note that 3 years after the beginning of the SARS-CoV-2 pandemic, and despite the availability of several vaccines for 2 years, the restrictive measures to contain SARS-CoV-2 spreading were met with several challenges among most countries. In particular, the recent variants are generating new outbreaks of infection, even in countries where the level of vaccinations is high. However, it becomes necessary for continuous monitoring of the most predominant SARS-CoV-2 lineages as well as their specific dynamic and processes of evolution. Therefore, this knowledge gain and continual analysis of variant lineages is imperative for epidemiologists to define public health measures, perform adequate diagnostic tests, and strategically employ vaccines (49).

Despite the number of positive cases of COVID-19 in Sergipe, these did not have minimum values to be submitted for genetic sequencing; and we were unable to establish a stratified correlation between the number of lineages and the severity of COVID-19 cases in both vaccinated and unvaccinated individuals. This correlation could have demonstrated how lineage variability impacts the severity of infections.

## Data availability statement

The original contributions presented in the study are included in the article/Supplementary material, further inquiries can be directed to the corresponding author.

## Ethics statement

Ethical approval was not required for the study involving humans in accordance with the local legislation and institutional requirements. Written informed consent to participate in this study was not required from the participants or the participants’ legal guardians/next of kin in accordance with the national legislation and the institutional requirements.

## Author contributions

MF and VQ: conceptualization. MF, KF, LS, FN, and JR: investigation. MF, KF, and ER: methodology. MF and KF: formal analysis. MF: project administration. MF and TM: writing – original draft. MF, TM, ML, MA, SS, KF, JR, LS, and CS: writing – review and editing. All authors contributed to the article and approved the submitted version.

## Footnotes

^1^ https://censo2022.ibge.gov.br/

^2^ https://www.gisaid.org/

^3^ https://cov-lineages.org/resources/pangolin.html

^4^ https://globalepidemics.org/

^5^ www.genomahcov.fiocruz.br/gisaid/

^7^ https://outbreak.info/compare-lineages
