# Supplementary figures and images for: The increase in SARS-CoV-2 lineages during 2020–2022 in a state in the Brazilian Northeast is associated with a number of cases
Source: Front Public Health. 2023 Dec 14;11:1222152. doi: 10.3389/fpubh.2023.1222152 (PMC10771345; doi:10.3389/fpubh.2023.1222152)

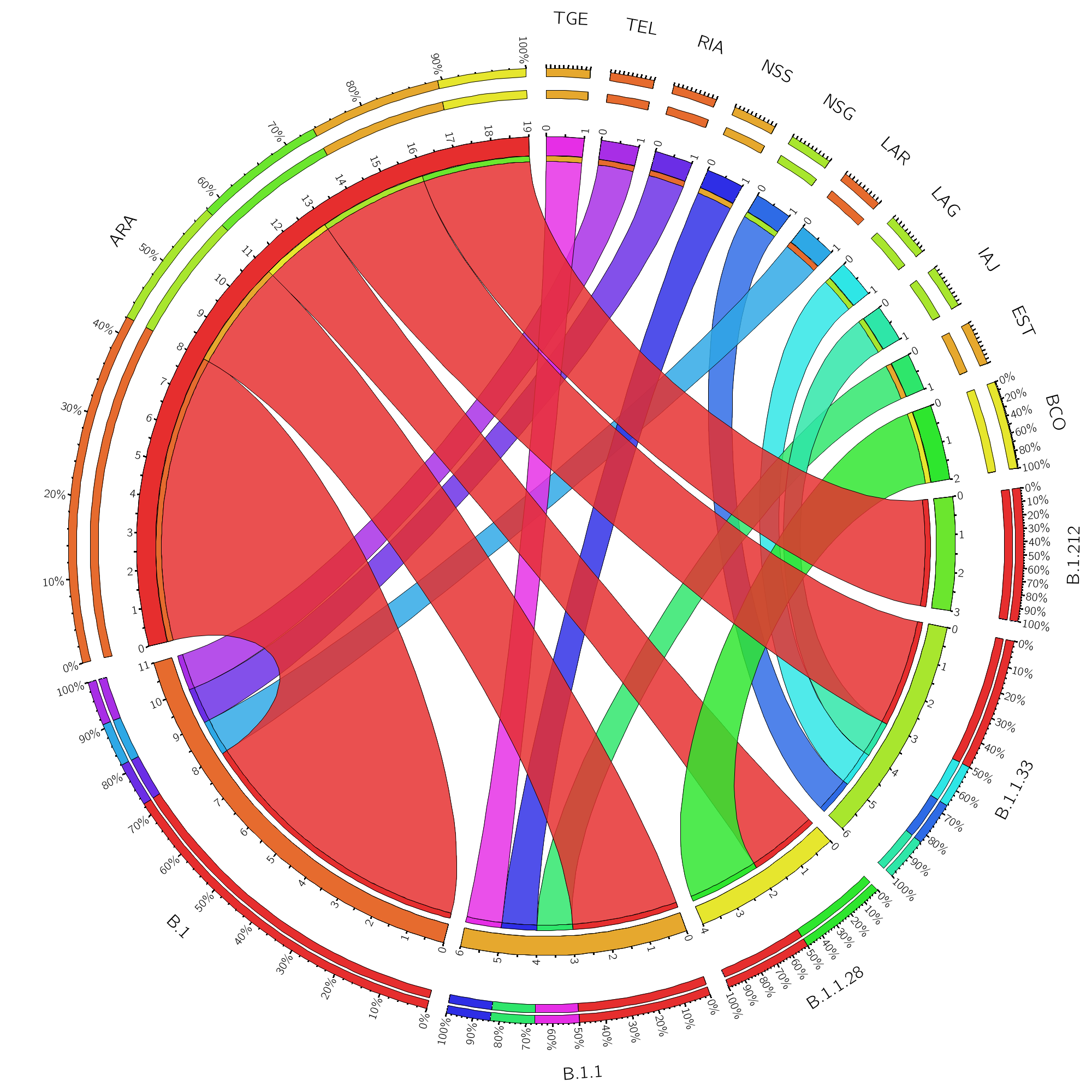

Supplement: Supplementary file 1 [file Image_1.TIF]

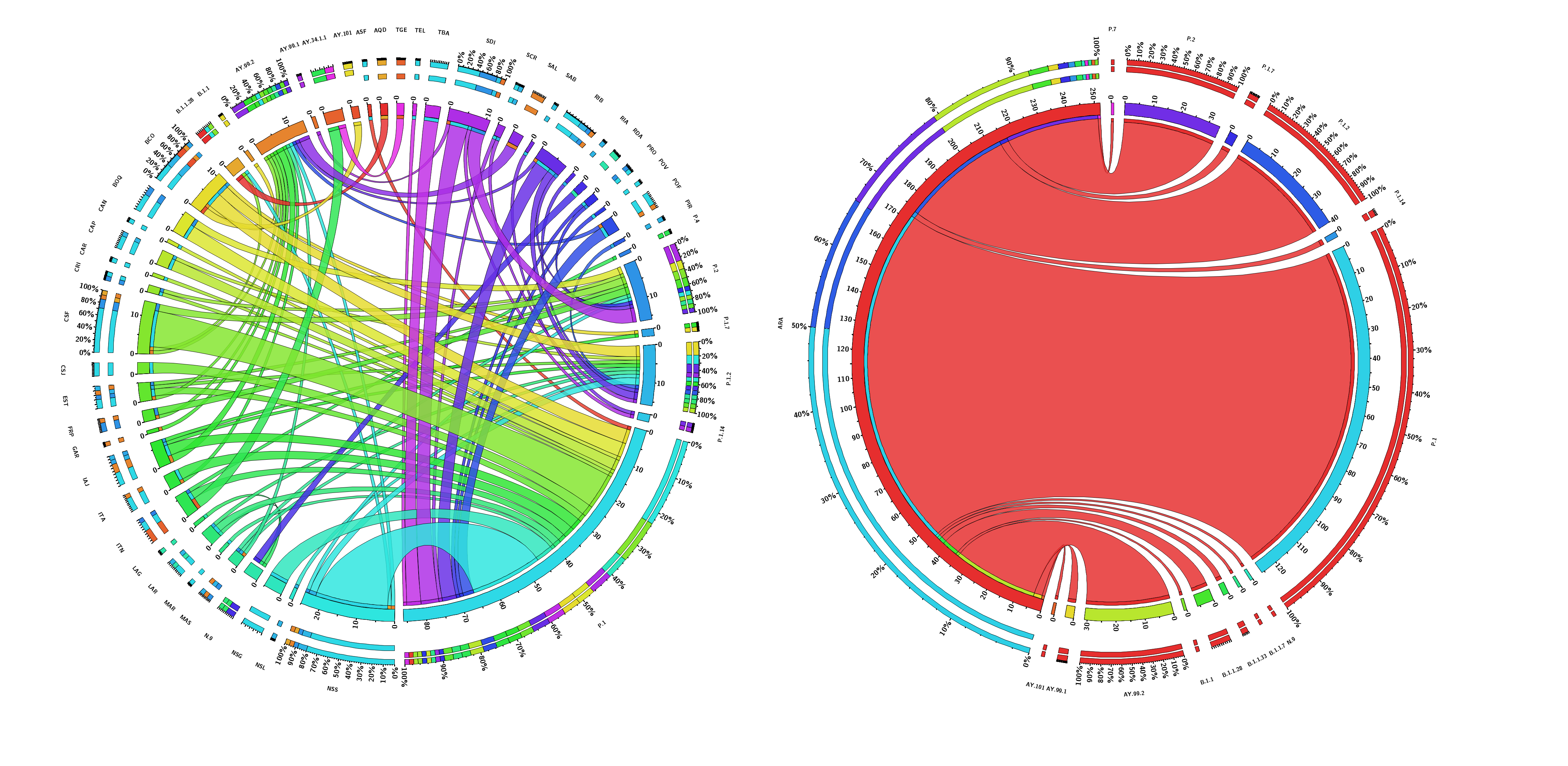

Supplement: Supplementary file 2 [file Image_2.TIF]

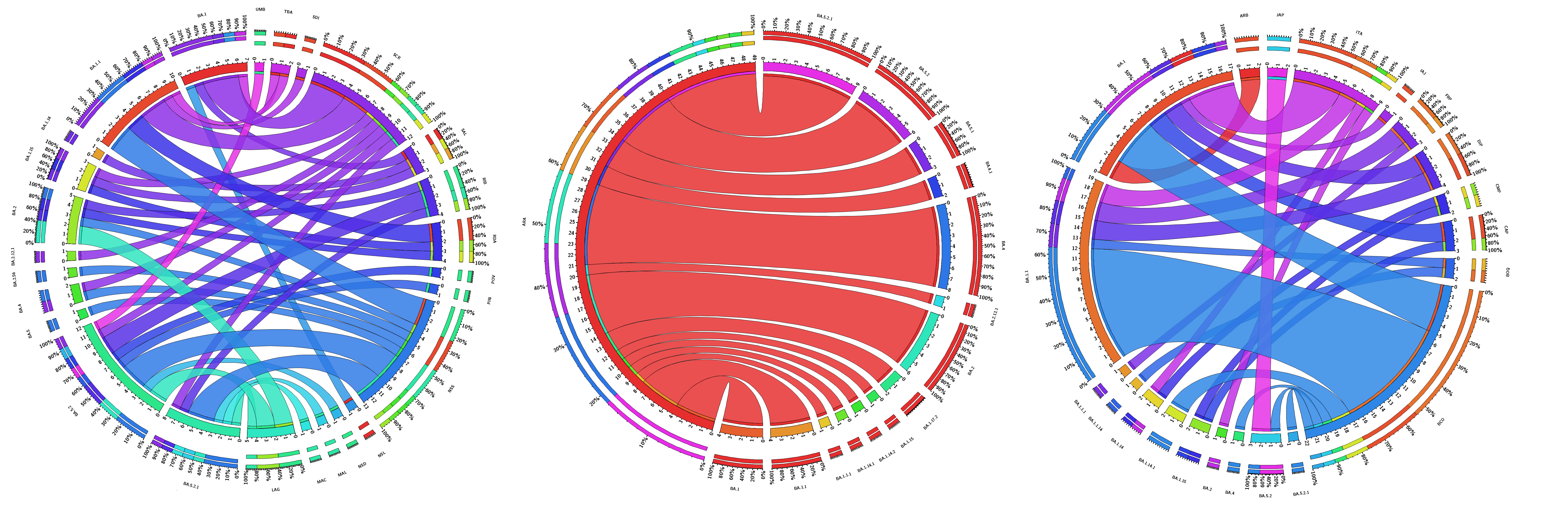

Supplement: Supplementary file 3 [file Image_3.TIF]
